# Venezuelan Equine Encephalitis Complex Alphavirus in Bats, French Guiana

**DOI:** 10.3201/eid2704.202676

**Published:** 2021-04

**Authors:** Carlo Fischer, Dominique Pontier, Ondine Filippi-Codaccioni, Jean-Batiste Pons, Ignacio Postigo-Hidalgo, Jeanne Duhayer, Sebastian Brünink, Jan Felix Drexler

**Affiliations:** Charité-Universitätsmedizin Berlin, corporate member of Freie Universität Berlin, Humboldt-Universität zu Berlin and Berlin Institute of Health, Institute of Virology, Berlin, Germany (C. Fischer, I. Postigo-Hidalgo, S. Brünink, J.F. Drexler);; Université de Lyon, Villeurbanne, France (D. Pontier, O. Filippi-Codaccioni, J.-B. Pons, J. Duhayer);; German Centre for Infection Research, Berlin, Germany (J.F. Drexler)

**Keywords:** Venezuelan equine encephalitis complex alphavirus, Tonate virus, bats, viruses, French Guiana, meningitis/encephalitis

## Abstract

Although essential for control strategies, knowledge about transmission cycles is limited for Venezuelan equine encephalitis complex alphaviruses (VEEVs). After testing 1,398 bats from French Guiana for alphaviruses, we identified and isolated a new strain of the encephalitogenic VEEV species Tonate virus (TONV). Bats may contribute to TONV spread in Latin America.

Venezuelan equine encephalitis complex alphaviruses (VEEVs) are arthropod-borne viruses (arboviruses) (*1*,*2*). Most VEE complex viruses can infect livestock and humans, causing predominantly acute febrile illness; some VEE complex viruses, including Tonate virus (TONV), which is the predominant VEE complex virus in French Guiana, also cause lethal encephalitis in humans (*3*). Clarifying the enzootic transmission cycles of VEEVs is essential for developing control strategies. Some VEEVs infect a broad range of invertebrate and vertebrate hosts, including horses, birds, rodents, and bats (*4*). Beyond humans, only birds have been identified as vertebrate hosts for TONV by direct virus detection and characterization (*5*,*6*). Bats are particularly relevant hosts of zoonotic viruses (*7*) and are potential hosts of selected VEEV subtypes in Mexico and Trinidad (*4*,*8*). To gain more insights into the ecology of VEEVs in French Guiana, we sampled bats and tested them for alphavirus infections using molecular, serologic, and cell-culture–based tools. 

## The Study

We screened serum samples from 1,398 individual animals representing 25 different bat species collected during 2010–2018 in French Guiana using a broadly reactive alphavirus-specific reverse transcription PCR (RT-PCR) for viral RNA (Table 1) (*9*). All animals were released unharmed after sampling.

**Table 1:** Bats tested for Tonate virus infection by PCR and PRNT, French Guiana*

Species	Animals screened by RT-PCR							PRNT screening, positive/tested
	2010	2011	2012	2015	2016	2017	Total	
*Anoura geoffroyi*	48	29	74	40	50	13	254	1/13
*Artibeus lituratus*	0	0	0	0	3	1	4	0
*A. obscurus*	0	0	0	0	2	8	10	0/10
*A. planirostris*	0	0	0	0	21	35	56	1/10
*Carollia perspicillata*	3	6	14	6	97	119	245	1/17
*Cynomops planirostris*	0	0	0	0	0	4	4	0
*Dermanura cinerea*	0	0	0	0	9	16	25	0
*Desmodus rotundus*	1	1	5	13	0	2	22	1/13
*Lonchorhina inusitata*	0	0	0	0	3	3	6	0
*Molossus molossus*	0	0	0	0	56	35	91	0/20
*M. rufus*	0	0	0	0	9	1	10	0
*Noctilio albiventris*	0	0	0	0	2	3	5	0
*N. leporinus*	0	0	0	0	1	25	26	0/20
*Phyllostomus latifolius*	5	1	2	1	0	0	9	0
*P. hastatus*	0	0	0	20	16	30	66	0
*Platyrrhinus brachycephalus*	0	0	0	0	3	8	11	0
*P. fusciventris*	0	0	0	0	0	9	9	0
*P. incarum*	0	0	0	0	3	1	4	0
*Pteronotus gymnonotus*	0	0	0	0	0	11	11	0
*Pteronotus* sp.	61	79	33	119	85	81	458	0/45
*Sturnira lilium*	0	0	0	0	6	20	26	0/8
*S. tildae*	0	0	0	0	17	8	25	0
*Tonatia saurophila*	1	0	0	0	0	3	4	0
***Trachops cirrhosus***	2	**1**	0	0	6	2	11	0/11
*Uroderma bilobatum*	0	0	0	0	2	4	6	0
Total	121	117	128	199	391	442	1398	4/167

*PCR-positive bat species and sample are highlighted in bold. was conducted using a screening dilution of 1:50 only because sample volumes were limited. PRNT, plaque reduction neutralization test; RT-PCR, reverse transcription PCR.

**Figure 1:**
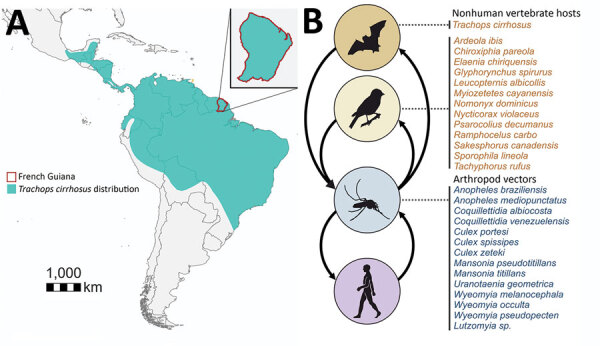
Tonate virus hosts and cycles for study of Venezuelan equine encephalitis complex alphavirus in bats, French Guiana. A) Geographic location of French Guiana in South America and distribution of fringe-lipped bats according to the International Union for Conservation of Nature Red List (https://www.iucnredlist.org/). B) Schematic transmission cycles of TONV according to data from this study and preliminary studies (*5*,*6*).

**Figure 2:**
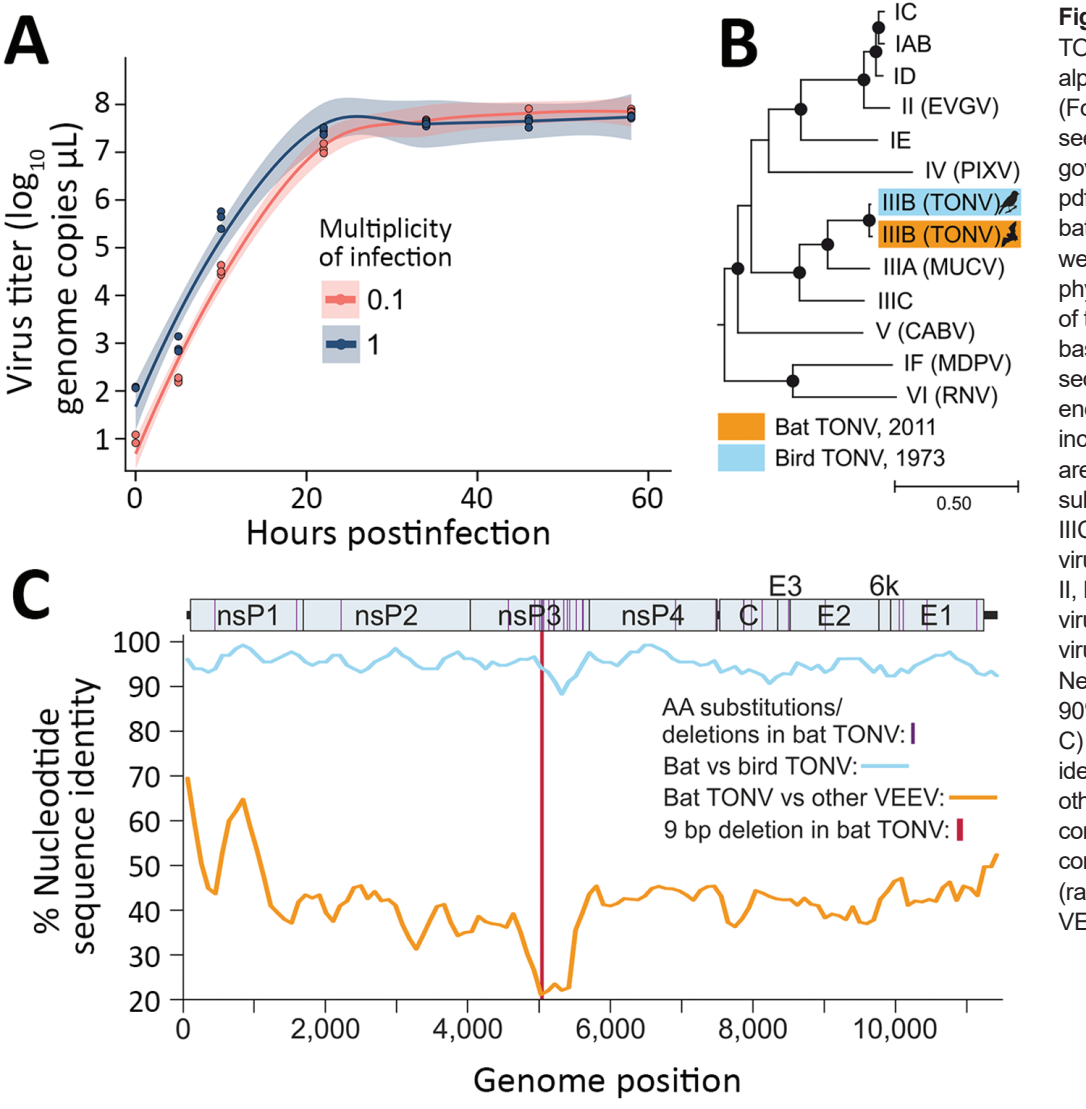
Characterization of bat TONV in study of VEE complex alphavirus in bats, French Guiana. (For additional discussion of methods, see Appendix.) A) Growth kinetics of the new bat TONV on Vero E6 cells in 24-well plates. (B) Maximum-likelihood phylogeny of TONV and members of the VEEV antigenic complex based on full genome nucleotide sequences. Eastern equine encephalitis virus (NC_003899) was included as an outgroup. Viruses are named according to the VEEV subtype classification: IAB/IC/ID/IE/IIIC, Venezuelan equine encephalitis virus; IF, Mosso das Pedras virus; II, Everglades virus; IIIA, Mucambo virus; IIIB, Tonate virus; IV, Pixuna virus; V, Cabassou virus; VI, Rio Negro virus. Bootstrap support above 90% is highlighted by filled circles. C) Percentage nucleotide sequence identity between TONV isolates and other viruses of the VEE antigenic complex. Consensus preparation was done using Geneious 9.1.8 (https://www.geneious.com) by mapping to the TONV CaAn 410d complete genome (GenBank accession no. NC 038675.1) as reference. The median coverage for consensus preparation was 5,504 (range 5–11,605). TONV, Tonate virus; VEE, Venezuelan equine encephalitis.

The overall TONV detection rate among all tested animals was 0.07% (95% CI −0.07% to 0.21%). Only 1 apparently healthy fringe-lipped bat (*Trachops cirrhosus*) sampled in 2011 was PCR-positive; we classified the virus as TONV (also known as VEEV subtype IIIB) upon amplicon sequencing (*6*). Among 11 individual fringe-lipped bats, the detection rate was 9.1% (95% CI −11.2% to 29.3%) (Figure 1, panel A). The TONV-positive sample was quantified by real-time RT-PCR using strain-specific oligonucleotides and an in vitro transcribed RNA standard (Table 2). Although the concentration of viral RNA in this sample was low, 78.5 genome copies/μL of blood, the virus was isolated on Vero E6 cells, suggesting potential to infect cells of primate origin. Successful isolation was consistent with highly efficient replication in cell culture, reaching 10^7^ copies/µL of supernatant within 24 hours at different multiplicities of infection (Figure 2, panel A).

**Table 2:** Oligonucleotides for quantification of TONV, French Guiana*

Name	Sequence, 5′ → 3′	Concentration
Forward primer	CATTGTCATAGCCAGCAGAGTTCT	400 nM
Reverse primer	GACTTGATACCTTTGACGATGTTGTC	400 nM
Probe (FAM-labeled)	CGCGAACGTCTGACCAACTCACCCT	200 nM

*We carried out 25 μL real-time RT-PCR reactions using the Superscript III one-step RT-PCR system with Platinum Taq polymerase (Thermo Fisher Scientific, https://www.thermofisher.com). Reactions were set up with 5 μL extracted RNA; 12.5 μL of 2× reaction buffer; 0.4 μL of a 50 mM magnesium sulfate solution; 1 μg of nonacetylated bovine serum albumin; and 1 μL enzyme. Amplification was conducted at 50°C for 15 min, followed by 95°C for 3 min and 45 cycles of 95°C for 15 s and 58°C for 30 s with fluorescence, read at the 58° annealing/extension step on a LightCycler 480 thermocycler (Roche, https://www.roche.com). FAM, fluorescein amidite; RT-PCR, reverse transcription PCR.

The complete viral genome was generated from the original isolate by high-throughput sequencing (MiSeq V3 chemistry; Illumina, https://www.illumina.com). In a complete genome-based maximum likelihood phylogeny, the bat-associated TONV (GenBank accession no. MW809725) clustered with the only available TONV strain, which was isolated in 1973 from a bird (Figure 2, panel B). Despite ≈40 years between the 2 TONV isolations and despite the divergent vertebrate hosts, the nucleotide identity between the bat-associated and the bird-associated TONVs was 98.1%, averaged over the whole genome. The high rate of genomic conservation is probably a consequence of purifying selection that is a predominant evolutionary force acting on arboviruses because of their need to infect both vertebrate and arthropod cells (*10*). Nucleotide identity was <90% only within the hypervariable region (HVR) located in the alphaviral genomic region encoding the nonstructural protein 3 (nsP3) (Figure 2, panel C). In total, 31 aa substitutions or deletions were present in the bat-associated TONV compared with the bird-associated TONV, of which 14 were located within the nsP3 HVR (p<0.0001 by χ^2^ test comparing the HVR to other genomic regions) (Figure 2, panel C). At the 5′ end of the HVR, the bat-associated TONV showed a larger in-frame deletion of 9 nt compared with the bird-associated TONV. This genomic region was covered by roughly 8,000 reads, supporting the deletion not being caused by technical mistakes during sequencing (Appendix Figure). The nsP3 HVR is assumed to play a crucial role for vector adaptation of VEEVs (*11*), supported by experimental evidence showing that exchanging the nsP3 of the alphaviruses chikungunya virus and o’nyong nyong virus dramatically affects the ability of chimeras to infect *Anopheles* and *Aedes* mosquito cells (*12*). The nsP3 deletion may therefore hypothetically reflect viral adaptation to different invertebrate, and potentially also vertebrate, hosts. Cell culture–based experiments including mosquito, bat, and bird cell lines, as well as in vivo infections, comparing the growth of both TONV isolates and chimeric viruses will be needed to yield definite assessments on the potential effect of the observed HVR deletion on the viral phenotype.

Detection of acute TONV infection in only 1 fringe-lipped bat was not surprising because alphaviral viremia is typically short-lived (*13*). To examine the frequency of past TONV infections in bats, we tested 167 bat serum samples for TONV-specific neutralizing antibodies by 50% plaque reduction neutralization test (PRNT_50_). We selected the sample set on the basis of availability of sufficient sample volumes; a preference for fringe-lipped bats, the attempt to represent the most abundant bat species investigated in this study; and a focus on bat genera in which VEEV-specific neutralizing antibodies had been detected previously in other countries (*4*,*8*). Four bats were seropositive, resulting in an overall TONV seroprevalence of 2.4% (95% CI 0.1%–4.7%) among tested samples. Limited reduction of PFUs at a serum dilution of 1:50 spoke against high antibody titers in those 4 animals and, indeed, no neutralization was observed when those 4 serum samples were tested at a dilution of 1:500. The 4 seropositive bats belonged to the species *Anoura geoffroyi* (1/13 animals, 7.7%; 95% CI −9.1% to 24.5%), *Artibeus planirostris* (1/10 animals, 10%; 95% CI −12.6% to 32.6%), *Carollia perspicillata* (1/17 animals, 5.9%; 95% CI −6.6% to 18.4%), and *Desmodus rotundus* (1/13 animals, 7.7%; 95% CI −9.1% to 24.5%). All 11 fringe-lipped bats, including the acutely infected PCR-positive animal, showed no detectable neutralization of TONV. 

Our serologic data are limited by testing only 1 relatively high serum dilution, and by the inability to differentiate between the neutralization of TONV and of other VEEVs such as Cabassou or Mucambo virus, which occur in geographic proximity to TONV (*14*). The serologic data therefore support low-level circulation of TONV or of antigenically related VEEVs in different bat species. Low prevalence of antibodies neutralizing TONV is consistent with the detection of VEEV antibodies in *Desmodus rotundus* (4.9%), *Carollia perspicillata* (6.9%), *Artibeus* spp. (4.4%), and *Noctilio leporinus* (7.1%) bats in Trinidad by epitope‐blocking ELISA and hemagglutination inhibition tests (*8*).

## Conclusion

The breadth of the VEEV host range remains unknown for most VEEV species or subtypes (*1*). This lack of information is particularly true for TONV, which has been found only in birds and humans so far. Identifying bats as naturally infected TONV hosts is thus a key finding, indicating a broad vertebrate host range for TONV. The TONV host range may hypothetically include other vertebrates, such as rodents, based on natural infections by other VEEVs closely related to TONV (*4*) and by preliminary data on TONV infections in sentinel mice in the 1970s (*5*) (Figure 1, panel B). In French Guiana, 12% of the overall human population shows serologic evidence for prior TONV infection, but the regions of highest risk for TONV infection remained unclear. One serosurvey reported highest seroprevalence in the coastal regions (35%) (*5*), whereas another serosurvey reported highest seroprevalence in inland savannah areas (53%) (*15*). The broad distribution of TONV might be explained by a broad vertebrate host range adding to the previously known broad invertebrate host range (*2*,*5*). Bats are extraordinary species and hosts for many zoonotic viruses and may thus also play a major role in TONV maintenance (*7*). Future research addressing TONV transmission cycles should include sampling of a broad range of vertebrate animals in ecologically different habitats, ideally including bats and analyses of TONV-competent mosquito bloodmeals.

**Appendix.** Additional information on Venezuelan equine encephalitis complex alphavirus in bats, French Guiana.
