# Supplementary material for: Venezuelan Equine Encephalitis Complex Alphavirus in Bats, French Guiana
Source: Emerg Infect Dis. 2021 Apr;27(4):1141–5. doi: 10.3201/eid2704.202676 (PMC8007291; doi:10.3201/eid2704.202676)
Supplement: Supplementary file 1 — Appendix. Additional information on Venezuelan equine encephalitis complex alphavirus in bats, French Guiana. [file 20-2676-Techapp-s1.pdf]

Article DOI: https://doi.org/10.3201/eid2704.202676

Venezuelan Equine Encephalitis Complex Alphavirus in Bats, French Guiana

Appendix

Additional Methods

For isolation, we seeded cells to 90% confluency in 24-well plates. We inoculated cells with 180 µL of 1:50 and 1:500 diluted serum for 1 hour. Afterward, we added 420 µL Dulbecco modified Eagle medium (DMEM) supplemented with 5% fetal calf serum, 1% penicillin/streptomycin (100 U/mL), and 1% nonessential amino acids to a final volume of 600 µL. Cells were incubated at 37°C and 5% CO_2 with daily controls for cytopathic effects. For infection, we replaced the growth medium by 200 µL DMEM (1% FCS) and added 50 µL diluted virus. After 1 hour we removed the inoculum and washed the cells 3 times using PBS. We added 1 mL fresh DMEM (10% FCS) to the cells and incubated the cells for 96 hours. We collected 50-µL samples regularly to analyze virus growth by real-time RT-PCR.

We used the maximum-likelihood method and a general time reversible model in MEGA-X (https://www.megasoftware.net), with a discrete gamma distribution to model evolutionary rate differences among sites and a complete deletion option. Statistical support of grouping was determined by 500 bootstrap replicates. For all viruses, we used the ICTV reference sequences (https://talk.ictvonline.org/ictv-reports/ictv_online_report/positive-sense-rna-viruses/w/togaviridae/872/genus-alphavirus). Identity was calculated using the International Committee on Taxonomy of Viruses reference sequences and SSE version 1.3 (http://www.virus-evolution.org/Downloads/Software/), with a fragment length of 400 and an increment between fragments of 100 residues. We extracted viral RNA using the QIAamp Viral RNA Mini Kit (QIAGEN, https://www.qiagen.com). Library preparation and Illumina MiSeq sequencing for full genome generation of the bat TONV was done using the KAPA Frag Kit, KAPA HyperPrep kit (Roche Molecular Diagnostics, https://diagnostics.roche.com), and MiSeq reagent v2 chemistry (Illumina, https://www.illumina.com).

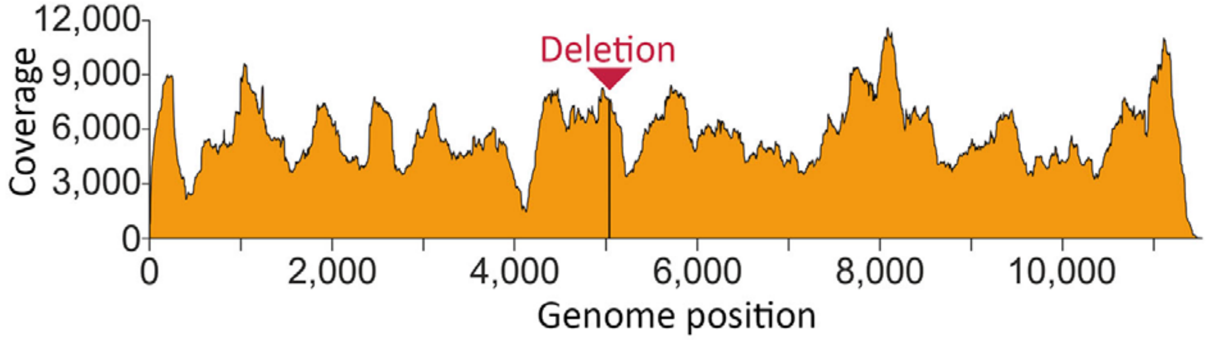

Appendix Figure. Genome coverage based on high-throughput sequencing reads mapped to the Tonate virus strain CaAn 410d complete genome (GenBank accession no. NC 038675.1). Mapping of reads was conducted using Geneious 9.1.8 (https://www.geneious.com). The 9 bp in-frame deletion at the 5′-end of the hypervariable region is highlighted.
